# Do type A personality and neuroticism moderate the relationships of occupational stressors, job satisfaction and burnout among Chinese older nurses? A cross-sectional survey

**DOI:** 10.1186/s12912-022-00865-7

**Published:** 2022-04-15

**Authors:** Mengxin Lu, Feng Zhang, Xiaohong Tang, Liping Wang, Jinling Zan, Yan Zhu, Danjun Feng

**Affiliations:** 1 School of Nursing and Rehabilitation, Shandong University, No. 44 Wenhuaxi Road, Jinan, 250012 Shandong China; 2 Department of Thoracic Surgery, Shandong Provincial Hospital, Jinan, China; 3 Peking University International Hospital, Peking, China; 4 People’s Hospital of Rizhao Lanshan, Rizhao, China; 5 Zaozhuang Municipal Hospital, Zaozhuang, China; 6 Zibo Central Hospital, Zibo, China

**Keywords:** Burnout, Job satisfaction, Neuroticism, Nurses, Occupational stress, Type A personality

## Abstract

**Background:**

The high prevalence of burnout among nurses produces huge health service losses. Existing literature found that occupational stressors and low levels of job satisfaction were related to burnout, whilst personality traits such as type A personality and neuroticism influenced occupational stressors, job satisfaction, and burnout. The purpose of this study was to investigate the mediating effect of job satisfaction on the relationship between occupational stressors and burnout among Chinese older nurses, and explore the moderating effects of type A personality and neuroticism on the relationships among occupational stressors, job satisfaction and burnout.

**Methods:**

A cross-sectional study was conducted in five provinces and municipalities (mainly in Shandong) in China. A total of 527 female older nurses (age≧40) were included in this study. Structural equation modelling (SEM) approach was employed to investigate the mediating effect of job satisfaction on the relationship between occupational stressors and burnout. Multi-group analysis was conducted to explore the moderating effects of type A personality and neuroticism on the relationships among occupational stressors, job satisfaction and burnout.

**Results:**

Both nurses with high type A personality and high neuroticism had higher occupational stressors, higher burnout and lower job satisfaction. Occupational stressors had direct effect (β = 0.29, *P* = 0.001) and indirect effect mediated by low levels of job satisfaction (β = 0.25, *P* = 0.001) on burnout. Type A personality had significant moderated effect (*P* = 0.007) on the relationships among occupational stressors, job satisfaction and burnout, whereas the moderated effect of neuroticism was not significant.

**Conclusions:**

Low levels of job satisfaction mediated the relationship between occupational stressors and burnout among Chinese older nurses, and both the direct and indirect effect of occupational stressors on burnout were moderated by type A personality. Hospital administrators should take specific measures such as transferring older nurses to easier positions to reduce their occupational stress, thereby increasing their job satisfaction and reducing their burnout, which is especially important for the older nurses with high type A personality.

**Supplementary Information:**

The online version contains supplementary material available at 10.1186/s12912-022-00865-7.

## Introduction

Burnout is a psychological syndrome caused by exposure to chronic work-related stressors [1]. It consists of three dimensions: emotional exhaustion, depersonalization, and reduced personal accomplishment [2]. It is well known that the nursing profession is characterized by complicated working conditions, heavy workloads, and relatively poor socioeconomic status [3]. Nurses are prone to burnout if they cannot cope well with these problems. Indeed, the literature confirms a higher prevalence of burnout among nurses than other professionals [4]. The high turnover rate and early retirement caused by burnout [5] leads to huge costs to the healthcare system and reduces the quality of care for patients [6]. In addition, burnout is associated with many adverse outcomes for nurses, such as insomnia, anxiety, depression, and social dysfunction [7]. Hence, burnout of nurses should be paid enough attention to.

According to Maslach et al. [2], determinants of burnout consist of two dimensions: situational and individual factors. Occupational stress is one of the most important situational factors. Chinese nurses usually undertake tasks beyond their duties, including patient transit, equipment maintenance, and routine urethral catheterization. Furthermore, they need to undertake the consequences of tense nurse-patient relationships and are vulnerable to medical disputes [8]. Therefore, Chinese nurses may experience more occupational stressors, which makes them more prone to suffer burnout [9].

As an individual factor influencing burnout, job satisfaction refers to an attitude toward one’s job, which arises from job experiences and makes individuals develop different degrees of burnout even when facing the same work situation [10]. It is widely observed that job satisfaction is strongly negatively correlated with burnout. For example, evidence from medical workers and geriatric care workers indicated that job satisfaction had a significant, negative influence on burnout [11, 12]. Furthermore, previous studies of nurses from Turkey [13] and South Africa [14] also confirmed that job satisfaction predicted lower levels of burnout. In addition, individuals with more occupational stress usually have low job satisfaction [15]. Some individual occupational stressors such as workload [16] or work environment [10] were also strongly correlated with low levels of job satisfaction. Based on the aforementioned studies, job satisfaction may play a mediating role in the relationship between occupational stressors and burnout among older nurses.

Personality is a sum of psychological characteristics that are relatively stable in adulthood, and it reflects one’s adaptability to the environment on the basis of unique behaviour and thinking patterns [17]. In the light of the differential exposure-reactivity model of the stress process, personality differences may affect both exposure and reactivity to stressful events [18]. Specifically, the individual’s perceptions of occupational stress and how they affect job satisfaction and burnout may be influenced by personality traits. In order to obtain a deep understanding of the burnout mechanism, it is necessary to explore the role of personality traits.

Type A personality is characterized by achievement striving, high job engagement, time urgency, competitiveness, impatience, and hostility [19]. Owing to the characteristics of type A personality, people with it tend to have higher burnout, more occupational stressors, and lower job satisfaction [20, 21]. Studies also reported that the type A personality’s penchant for achievement striving was related to lower exhaustion and higher job satisfaction, whereas its impatience and hostility were related to higher exhaustion and lower job satisfaction [22, 23]. The different effects of the underlying components of type A personality on burnout and job satisfaction suggested that our mediated model might vary among different levels of type A personality.

Neuroticism characterized by emotional instability, negative emotional response, and stress sensitivity is usually robustly correlated with adverse health outcomes [24]. Individuals with high neuroticism had greater exposure and reactivity to stressors [18]. For example, studies indicated that neuroticism was positively related to perceived stress [25] and burnout [26], and negatively related to job satisfaction [27]. Additionally, existing studies found that people with high neuroticism perceived more work-related stress [28] and experienced stronger burnout [17] when experiencing similar occupational stressors as people with low neuroticism. Thereby, the mediation model may also differ across the different levels of neuroticism.

It is important to note that most studies on burnout focus on new graduate nurses because of their susceptibility to burnout [29]; however, burnout among older nurses also deserves attention. On the one hand, older nurses are the backbone of a hospital because they have abundant clinical experience and improved nursing skills [30]. Some of them are also clinical nursing teachers or head nurses who take responsibility for teaching or managing younger nurses, which suggests that burnout of older nurses will severely affect them and patients, as well as younger nurses. On the other hand, older nurses constitute an increasing proportion of the nursing workforce due to the worldwide aging workforce [30], and it is crucial to retain them to ensure adequate nursing human resources in light of the global shortage of nurses. Reducing burnout may be an effective measure to retain older nurses [5]. Therefore, the current study focused on burnout among older nurses. Considering that the legal retirement age in China (male 60, female 55) is lower than that in developed countries (65 and above), and referring to other studies [31], we set the older nurses as those over 40 years old.

**Fig.1:**
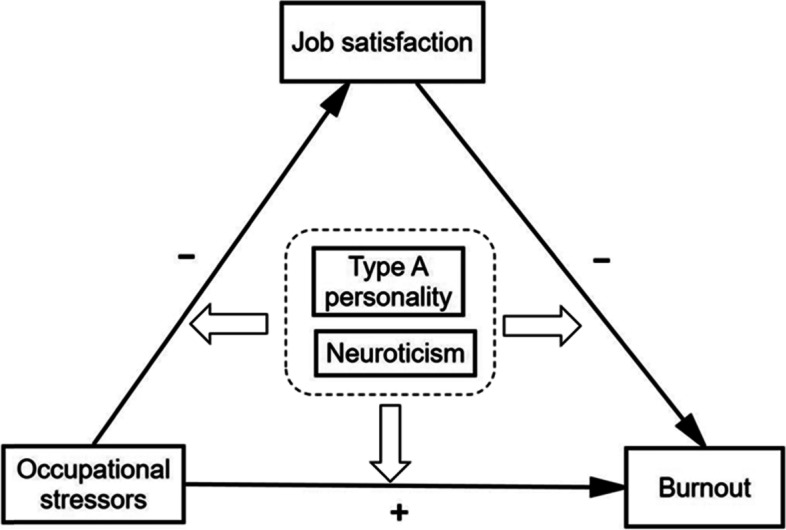
Hypothesized model of relationships among occupational stressors, job satisfaction, burnout, type A personality and neuroticism

In summary, there are two purposes in this study. The first is to examine the mediating role of job satisfaction in the relationship between occupational stressors and burnout. The second aim is to explore the moderating effect of type A personality and neuroticism on this mediated model. We hypothesized that occupational stressors can lead to burnout not only directly but also indirectly through low levels of job satisfaction. Furthermore, we assumed that the mediation model was different across different type A personality groups as well as across different neuroticism groups. The hypothetical model we developed is shown in Fig. 1.

## Methods

### Participants and procedure

This was a descriptive cross-sectional study conducted in 10 hospitals in five provinces and municipalities (Shandong, Beijing, Jilin, Liaoning, and Guangdong) in China. A convenience sampling method was used to recruit hospital nurses over 40 years old from March 2018 to October 2018. The inclusion criteria were as follows: (1) nurses with a “Nurse Professional Qualification Certificate”; (2) aged ≥ 40 years; and (3) volunteer to participate in this study. The exclusion criteria were: (1) nurses who were undergoing continuing education or training; and (2) nurses who were on vacation or retired during the survey period. In Jinan, Shandong Province, where the researchers are located, we distributed paper questionnaires for participants to fill out on site; in other cities, we conducted online survey through a WeChat link. Before starting the survey, participants were provided with a statement detailing the purpose and methods of the study, the voluntary nature of participation, and the confidentiality of responses. The researchers also stated that the completion and return of the questionnaires would be regarded as consent to participate. Each participant received a gift (for the paper survey) or WeChat Lucky Money (for the online survey) after they filled out the questionnaires. Ethical approval was provided by the ethics committee of the School of Nursing and Rehabilitation at Shandong University (No. 2016-R-25). A total of 535 valid questionnaires were received, of which only eight questionnaires were from the male nurses and excluded from the analysis due to the lack of representation. Ultimately, 527 female nurses were included in this study.

### Measures

#### Burnout

Burnout was assessed with the revised Chinese version of Maslach Burnout Inventory—General Survey (MBI-GS) [32]. It consisted of three dimensions: emotional exhaustion (EE; 5 items), depersonalization (DE; 4 items), and personal accomplishment (PA; 6 items). The items were rated on a 5-point scale from 1 (never) to 5 (everyday), with higher EE and DE subscales scores and lower PA subscale scores indicating a higher level of burnout. Confirmatory factor analysis (CFA) was used to examine the construct validity of the scale. We found that the first-order factor, “reduced personal accomplishment” had very low factor loading (0.27; the cutoff point is 0.4) [33] on burnout (the second-order factor); thus, it was deleted in the final analysis. Finally, only two dimensions remained in the MBI-GS (all factor loadings were above 0.73). The Cronbach’s α of emotional exhaustion, depersonalization, and total instrument was 0.92, 0.91 and 0.92, respectively.

#### Occupational stress

A revised Occupational Role Questionnaire of Occupational Stress Inventory [34] was used to examine the occupational stress. Initially, the inventory had four dimensions: role overload (6 items), role boundary (5 items), responsibility (6 items), and physical environment (5 items). The items were rated on a 5-point scale ranging from 1 (never) to 5 (often). Higher scores indicated more occupational stress. After CFA, we found the item “If I make a mistake in my work, it will lead to serious adverse consequences for others” had low factor loading (0.35) on responsibility (the first-order factor). Consequently, we deleted this item. Furthermore, as the first-order factor, physical environment had very low factor loading (0.29) on occupational stress (the second-order factor); therefore, we deleted the physical environment dimension. Finally, the inventory measured three aspects of occupational stress: role overload, role boundary, and responsibility. The inventory had good internal consistency (Cronbach’s α = 0.90) and construct validity (the lowest factor loading was 0.54).

#### Job satisfaction

A questionnaire was developed to measure job satisfaction, which included three items: (1) Overall, I am very satisfied with my job; (2) I regret doing this job; and (3) I would take the same job if given the chance to choose again. The items were answered on a five-point scale from 1 (strongly disagree) to 5 (strongly agree). Item 2 was reverse scored and added to the other items, with higher total scores indicating higher job satisfaction. The results of CFA showed that the questionnaire had good construct validity with factor loading no less than 0.62. The Cronbach’s α was 0.74.

#### Type A personality

Considering the uniqueness of Chinese culture, a questionnaire was developed to measure type A personality (see Additional file 1) that drew from the existing type A personality questionnaires used in Western countries, such as the MMPI-2 Type A Scale [35] and the Simplified Type A Questionnaire [36]. The original questionnaire had 22 items with a three-point scale ranging from 1 (strongly disagree) to 3 (strongly agree). A higher score indicated more type A personality traits. In order to explore and confirm the structure of type A personality, we conducted exploratory factor analysis (EFA) and CFA (each of them was based on nearly half of the sample). Ultimately, the questionnaire had four dimensions: time urgency and impatience (6 items), hostility and anger (7 items), competitiveness (2 items), and job engagement (2 items). This questionnaire explained 47.18% of the total variance in EFA and had good factor loading (no less than 0.44) in CFA. The Cronbach’s alpha for the questionnaire was 0.81.

#### Neuroticism

The neuroticism subscale from the Chinese version of the 44-item Big Five Inventory (BFI) [37] was applied to measure neuroticism. It consisted of 8 items with a five-point scale ranging from 1 (strongly disagree) to 5 (strongly agree). A higher score represented a higher level of neuroticism. The questionnaire had good construct validity (factor loading above 0.42) and reliability (Cronbach’s alpha = 0.81).

### Data analysis

SPSS 22.0 was used for data analysis. Descriptive statistics were performed to describe the socio-demographic characteristics of the participants as well as the scores of the study variables. Pearson correlation analysis was conducted to examine the correlations between the main variables. Confirmatory factor analysis (CFA) was conducted to verify the construct validity of questionnaires using Analysis of Moment Structure (Amos) 22.0.

The structural equation modelling (SEM) approach with the bias-corrected bootstrap method (2,000 replicates) was employed to explore the relationships between occupational stress, job satisfaction, and burnout (including direct effects and mediation effects) using Amos 22.0. The effect was significant if the bias-corrected bootstrap 95% confidence interval (CI) did not include “0”. To assess the goodness of fit of each model, a range of model-fit indices were reported: χ^2^/df (values < 3), root mean square error of approximation (RMSEA, values < 0.05), the goodness-of-fit index (GFI, values > 0.90), comparative fit index (CFI, values > 0.90), and Tucker–Lewis index (TLI, values > 0.90) [38].

The median split method was used to dichotomize type A personality traits (Median = 1.88) into high and low type A personality group. Neuroticism (Median = 2.63) was divided into high-level group and low-level group in the same way. Multi-group analysis was conducted to compare the differences in the relationships between occupational stress, job satisfaction, and burnout across high and low type A personality/neuroticism groups. This was done by comparing the differences of goodness-of-fit statistics from unconstrained model, partially constrained model to fully constrained model [39].

## Results

There were 527 female nurses participating in this study. The socio-demographic characteristics were shown in Table 1.

**Table 1:** Socio-demographic of the 527 participating nurses

Variables	*(M* ± *SD) or N* (%)
Age	46.15 ± 4.63
Years of work	25.18 ± 5.57
Province or municipality	
Shandong	395(75.0)
Beijing	89(16.9)
Jilin	22(4.2)
Liaoning	10(1.9)
Guangdong	11(2.1)
Marriage	
Married	503(95.4)
Single/divorced/widowed	19(3.6)
Miss value	5(0.9)
Education	
High school education or below	29(5.5)
Associate degree	124(23.5)
Bachelor’s degree	350(66.4)
Master’s degree or PhD	18(3.4)
Miss value	6(1.1)
Job title	
Nurse Practitioner	52(9.9)
Nurse-in-charge	374(71.0)
Deputy chief /chief nurse	84(15.9)
Miss value	17(3.2)
Perceived economic situation	
Poor	116(22.0)
Moderate	368(69.8)
Good	43(8.2)
Type A personality	
Low	286(54.3)
High	241(45.7)
Neuroticism	
Low	278(52.8)
High	249(47.2)

*M* Mean, *SD* Standard Deviation

Occupational stress had positive correlation with burnout and negative correlation with job satisfaction, whereas job satisfaction was negatively associated with burnout. Furthermore, both type A personality and neuroticism were positively associated with occupational stress and burnout, and were negatively associated with job satisfaction. The means, SDs, and bivariate correlations of the variables assessed were presented in Table 2.

**Table 2:** Means, standard deviations and correlations of main variables

	M	SD	1	2	3	4	5
1.Type A personality	1.87	0.32	1				
2.Neuroticism	2.63	0.75	.40^***^	1			
3.Occupational stress	2.46	0.70	.35^***^	.20^***^	1		
4.Job satisfaction	3.22	0.98	-.16^***^	-.24^***^	-.15^***^	1	
5.Burnout	2.46	0.89	.26^***^	.37^***^	.38^***^	-.56^***^	1

*M* Mean, *SD* Standard Deviation

^***^P ≤ 0.001

**Fig. 2:**
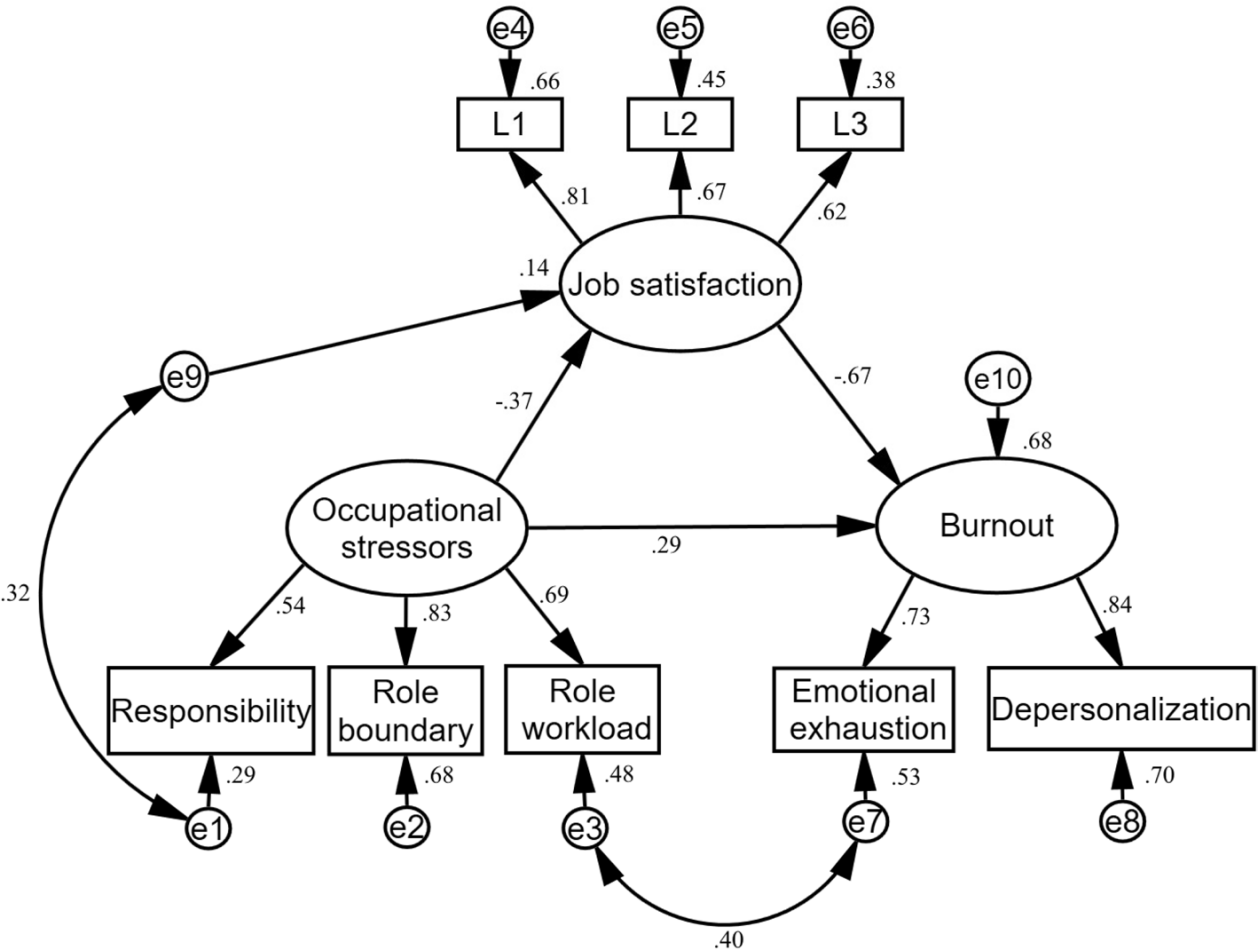
SEM analysis results of the mediation model. The variables named L1-L3 are the items of job satisfaction scale. All the coefficients in the figure are standardized and significant at 0.001 level

The SEM analysis showed that the initial indices of fit in the primary model were χ^2^/df = 7.38, RMSEA = 0.11, GFI = 0.94 CFI = 0.92, TLI = 0.87, and the data failed to support the theoretical model. In order to obtain an acceptable model fit, two pairs of error terms were correlated (see Fig. 2) according to the modification indices and theoretical justifications. Finally, we got a well-fitted model with χ^2^/df = 2.50, RMSEA = 0.05, GFI = 0.98, CFI = 0.98, TLI = 0.97. Results of the SEM analysis presented that occupational stress had a direct effect on burnout (see Fig. 2). Moreover, occupational stress also had a significant indirect effect on burnout (β = 0.25, *P* < 0.001) through low job satisfaction because higher occupational stress was associated with lower job satisfaction, which, in turn, was linked to higher burnout. Finally, this model explained 68% variance of burnout.

**Fig. 3:**
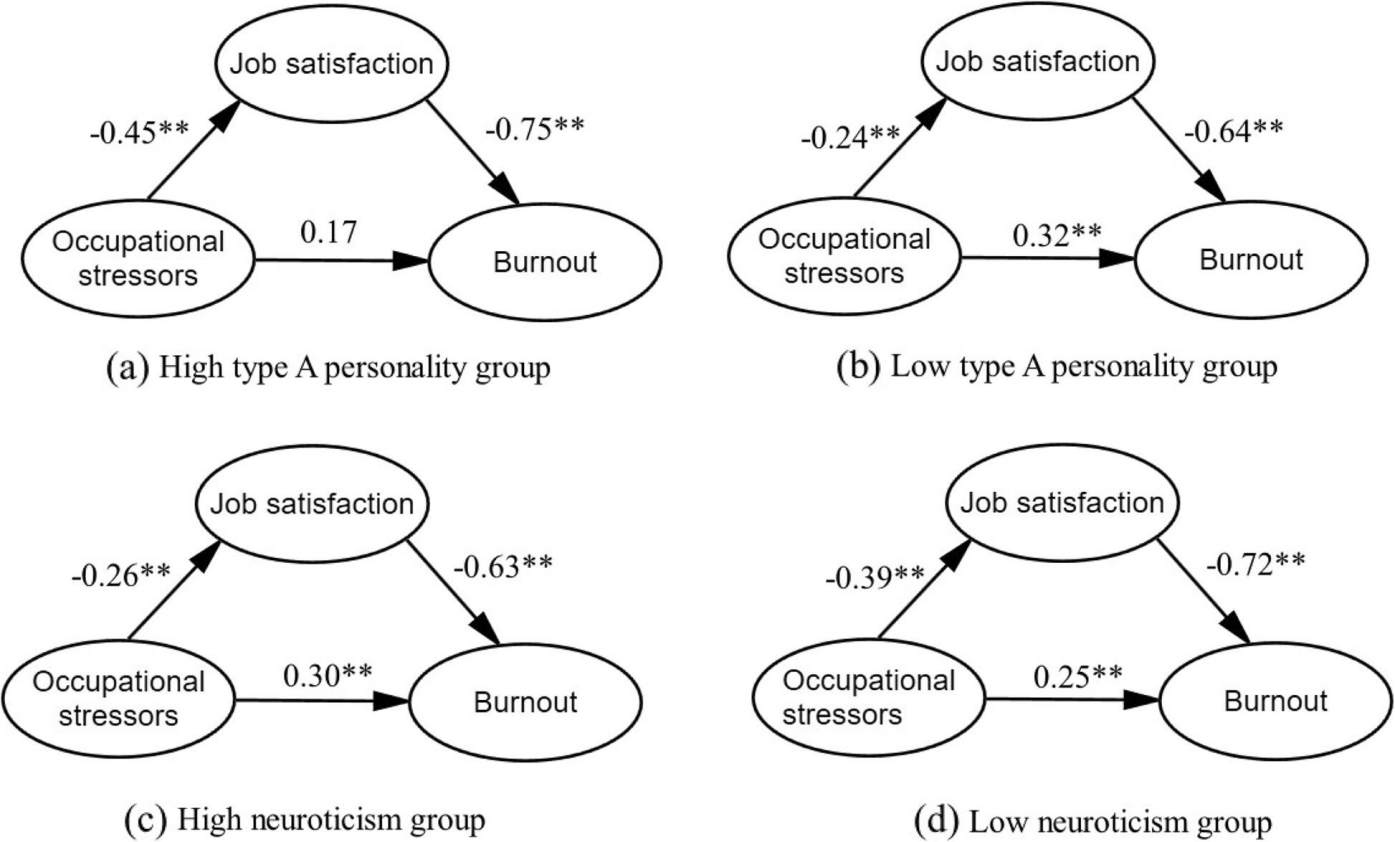
SEM analysis results of mediation model in different personality trait groups. **P ≤ 0.01

The results of multi-group analysis of the mediated model based on different personality groups were presented in Fig. 3. The result in type A personality groups showed that there was significant difference in goodness-of-fit statistics (*P* = 0.007) between the model with “restricted structural covariance” and the model with “restricted structural weights” (see Table 3), which indicated that the mediated model was significantly different for high and low type A personality. In the high type A personality group, there was a significant indirect effect (*P* = 0.001) mediated by job satisfaction, and a non-significant direct effect (*P* = 0.06) between occupational stressors and burnout. But both the indirect effect (*P* = 0.001) and direct effect (*P* = 0.001) were significant in the low type A personality group. The percentage of mediation effect in total effect among high type A personality group (66.00%) was almost twice that of the low type A personality group (33.33%). More details were shown in Table 4.

**Table 3:** Model comparison for the multi-group analysis based on different type A personality and neuroticism groups

Goodness-of-fit statistics	χ^2^(*df*)	△χ^2^(*df*)	*P*	GFI	CFI	TLI	RMSEA
**Type A personality groups**							
Model with no restrictions	51.24(30)			.98	.98	.97	.04
Model with restricted measurement weights	57.96(35)	6.73(5)	.242	.97	.98	.97	.04
Model with restricted structural weights	62.60(38)	4.64(3)	.200	.97	.98	.97	.04
Model with restricted structural covariance	69.86(39)	7.26(1)	**.007**	.97	.98	.97	.04
Model with restricted structural residuals	70.04(41)	0.18(2)	.916	.97	.98	.97	.04
Model with restricted measurement residuals	98.60(51)	28.57(10)	**.001**	.96	.96	.96	.04
**Neuroticism groups**							
Model with no restrictions	48.28(30)			.98	.99	.97	.03
Model with restricted measurement weights	58.13(35)	9.86(5)	.079	.97	.98	.97	.04
Model with restricted structural weights	62.32(38)	4.18(3)	.242	.97	.98	.97	.04
Model with restricted structural covariance	63.39(39)	1.08(1)	.299	.97	.98	.97	.04
Model with restricted structural residuals	71.11(41)	7.71(2)	**.021**	.97	.98	.97	.04
Model with restricted measurement residuals	87.29(51)	16.18(10)	.095	.96	.97	.97	.04

*GFI* Goodness of Fit Index, *CFI* Comparative Fit Index, *TLI* Tucker-Lewis Index, *RMSEA* Root Mean Square Error of Approximation

**Table 4:** The point estimates and 95% CIs for multi-group analysis of the mediation model

Groups	Indirect effects Estimate (95% CI)	Direct effects Estimate (95% CI)	Total effects Estimate (95% CI)	Mediation proportion
High type A personality group	.33 (.21, .49) ^**^	.17(-.01, .32)	.50 (.34, .64) ^**^	66.0%
Low type A personality group	.16 (.06, .27) ^**^	.32(.15, .47) ^**^	.48 (.32, .61) ^**^	33.3%
High neuroticism group	.16 (.04, .27) ^*^	.30 (.17, .44) ^**^	.47 (.29, .61) ^**^	34.0%
Low neuroticism group	.28 (.17, .42) ^**^	.25 (.08, .40) ^**^	.52 (.37, .65) ^**^	53.9%

***Note:*** All the above results were from unrestricted model. Total effects represented the total effects of occupational stress on burnout, which included direct effects and indirect effects. Direct effects represented the direct effects of occupational stress on burnout. Indirect effects represented the mediating effects of job satisfaction on the relationships between occupational stress and burnout. Mediation proportion = Indirect effects**/** Total effects

*CI* Confidence interval

^*^ *P* ≤ 0.05, ^**^P ≤ 0.01

The multi-group analysis results for neuroticism groups showed that the moderate effect of neuroticism on the mediated model was not significant. Although a significant difference of goodness-of-fit statistics between the model with “restricted structural residuals” and the model with “restricted structural covariance” was found (*P* = 0.021; see Table 3), the residual level differences should not impact the stability of the model across groups because the model of multi-group analysis is stable for measurement weights, structural weights and structural covariance. There was a significant indirect effect (*P* = 0.001) mediated by job satisfaction and direct effect (*P* = 0.001) between occupational stressors and burnout in both high and low neuroticism groups (see Fig. 3). However, the percentage of mediation effect in total effect among low neuroticism group (53.85%) was roughly 1.5 times that of high neuroticism group (34.04%). More details were presented in Table 4.

## Discussion

The existing literature reported that job satisfaction mediated the relationship between occupational stressors and burnout, but this relationship was not verified among older Chinese nurses, who may experience more occupational stressors than nurses in Western countries and cannot be replaced by young nurses. More importantly, to the best of our knowledge, this study is the first to explore the moderating effect of type A personality and neuroticism on the relationships between occupational stressors, job satisfaction and burnout.

The results of current study indicated that occupational stress not only directly led to burnout but also indirectly increased burnout by reducing job satisfaction among Chinese older nurses, which confirmed previous studies among American physicians [40] and Chinese banking system staff [41]. In order to alleviate their burnout, nursing administrators should take some measures to eliminate occupational stressors and increase job satisfaction. For instance, hospital administrators could recruit more nurses and clarify the scope of job responsibilities to reduce occupational stressors. Additionally, older nurses should be transferred to easier positions such as medical supply departments and medical examination centers. Furthermore, enhancing support from colleagues and organizations, providing professional training, and instituting fair promotion opportunities are also important to improving job satisfaction.

The most important purpose of present study was to explore the moderating role of type A personality and neuroticism in this mediated model. In our study, the mediation model was significantly different across type A personality groups. The older nurses with high type A personality had higher burnout, which could be explained by the mediation model in the current study. The mediation model showed that higher occupational stress led to lower job satisfaction, which contributed to higher burnout. The older nurses with high type A personality are ambitious and are prone to undertake more tasks such as clinical management and teaching besides daily nursing work to satisfy their sense of accomplishment, which undoubtedly could increase their workload. Furthermore, the hostile and impatience traits make older nurses with type A personality more likely to have interpersonal conflicts with other people (e.g., workmates and patients), which could additionally increase their occupational stress and thus reduce their job satisfaction. Given the high occupational stress and low job satisfaction, older nurses with high type A personality have higher levels of burnout. In the low type A personality group, occupational stress could directly increase burnout and indirectly increase burnout by reducing job satisfaction. However, in the high type A personality group, the association between occupational stress and burnout was totally mediated by job satisfaction. The results may partially be attributed to the achievement striving, and the impatience/hostility of type A personality [42]. In general, individuals with high occupational stress are inclined to develop burnout [43]. However, when placed in stressful situations, high type A individuals work hard to pursue achievement and then suppress their feelings of fatigue, which was closely linked to burnout [44]. Due to the characteristic of achievement striving, the high occupational stress for nurses with high type A personality may not be entirely harmful [20]. Hence, type A personality buffered the direct negative effect of occupational stress on burnout to some extent, which could explain the insignificant direct effect of occupational stress on burnout among nurses with high type A personality. Unfortunately, occupational stress may also stimulate the impatience and hostility of nurses with high type A personality, which may make them experience more workplace conflicts [45], receive less social support from colleagues and superiors, and increase the likelihood that they will be unsatisfied with their work. Furthermore, high type A personality individuals are more inclined to exhibit frustration in response to high occupational stress, which also contributes to lower job satisfaction [46]. Therefore, the lower job satisfaction of nurses with high A personality experiencing the same occupational stress could explain the stronger mediating effect of job satisfaction on the relationship between occupational stress and burnout.

In contrast, the moderating role of neuroticism was not supported. In both the high and low neuroticism group, occupational stress directly led to burnout and indirectly increased burnout by reducing job satisfaction. Although the moderating effect on neuroticism was not significant, the percentage of mediation effect in total effect for the low neuroticism group was obviously higher than that of high neuroticism group (roughly 1.5 times), which suggested that the effect of occupational stress on burnout was more likely to be mediated by job satisfaction in the low neuroticism group, while occupational stress was more likely to directly increase burnout in the high neuroticism group. The insignificant moderating effect may be owing to the limited sampling range of this study, thus further studies in more representative samples are recommended to verify this moderating role of neuroticism.

These results deepened our understanding of the complex mechanism of personality traits influencing the relationships between occupational stressors, job satisfaction, and burnout. For all older nurses, measures to reduce occupational stressors and improve job satisfaction should be implemented to prevent burnout. Furthermore, it is essential to identify high-risk personality traits for burnout and take specific measures. For instance, the high type A personality and high neuroticism groups experienced higher burnout, higher occupational stressors and lower job satisfaction in this study. For nurses with high type A personality, job satisfaction fully mediated the relationship between occupational stressors and burnout, therefore it is more urgent for hospital managers to take effective measures to improve their job satisfaction. In the high neuroticism group, the direct effect of occupational stressors on burnout was stronger than the indirect effect mediated by job satisfaction; hence measures to reduce occupational stressors may be more effective.

In addition, unlike the commonly used three-dimension MBI-GS questionnaire, this study removed the reduced personal accomplishment dimension of burnout after CFA, which was consistent with one previous study that found the reduced personal accomplishment dimension did not fit in the burnout construct [47]. Maybe many people lack a sense of accomplishment at their job even if they do not present burnout, because they may just regard work as a means of making a living rather than a path toward self-actualization. Therefore, reduced personal accomplishment may not be an effective indicator to measure the level of burnout. Two pairs of covariance parameters were added in this study. This is acceptable because the model modification was supported by strong theoretical justifications. The correlation of error terms between “Responsibility” and “Job satisfaction” provided an example: people who scored higher in conscientiousness on the Big Five Personality Test may take on more responsibility and have higher job satisfaction [48]; therefore, the correlation of the covariance parameter is theoretically reasonable.

There are several limitations to the present study. First, convenience sampling was used in this study, which limited the generalization of results and the establishment of causality. Future studies should examine the relationships among these variables, especially the moderating effect of type A personality and neuroticism on this mediated model, in a more representative sample by adopting more scientific sampling methods such as stratified random sampling. Another limitation is that only female nurses were included in this study. Previous studies have found gender differences in job satisfaction between men and women [49, 50], so future studies should include both male and female samples to validate the stability of the proposed model in this study, as well as to test whether the mediation model differs across gender groups. Finally, despite the high reliability and validity of the instrument in this study, recall bias may be present due to the use of self-report questionnaires.

## Conclusions

In summary, this study demonstrated the mediating role of low levels of job satisfaction between occupational stress and burnout and the moderating role of type A personality on this mediating model. To reduce burnout among older nurses, nursing administrators should take measures such as transferring older nurses to easier jobs to reduce their occupational stress and increase their job satisfaction. In addition, hospital administrators should give more attention to older nurses with high type A personality because of their high occupational stress, low job satisfaction, and high burnout.

## Supplementary Information

**Additional file 1. **English and Chinese items of the Chinese type A personality inventory.

## Data Availability

The datasets used and/or analysed during the current study are available from the corresponding author on reasonable request.

## Abbreviations

SEM: Structural equation modelling
EFA: Exploratory factor analysis
MBI-GS: Maslach Burnout Inventory—General Survey
CFA: Confirmatory factor analysis
BFI: Big Five Inventory
CI: Confidence interval
RMSEA: Root mean square error of approximation
GFI: Goodness-of-fit index
CFI: Comparative fit index
TLI: Tucker–Lewis index
M: Mean
SD: Standard deviation
AMOS: Analysis of Moment Structure
EE: Emotional exhaustion
DE: Depersonalization
PA: Personal accomplishment
