# Supplementary material for: Do type A personality and neuroticism moderate the relationships of occupational stressors, job satisfaction and burnout among Chinese older nurses? A cross-sectional survey
Source: BMC Nurs. 2022 Apr 15;21:88. doi: 10.1186/s12912-022-00865-7 (PMC9013170; doi:10.1186/s12912-022-00865-7)
Supplement: Supplementary file 1 — Additional file 1. English and Chinese items of the Chinese type A personality inventory. [file 12912_2022_865_MOESM1_ESM.pdf]

Supplementary material

Additional file 1. English and Chinese items of the Chinese type A personality inventory

Items	strongly disagree 完全不同意	partly agree 部分同意	strongly agree 完全同意
1. I am always in a hurry, even if there's nothing urgent to deal with.<br>1.即使没有什么急事，我也总是匆匆忙忙。	1	2	3
2. I always want to finish a task as soon as possible.<br>2.我总想尽快完成一件事情。	1	2	3
3. I am always anxious when I have to wait.<br>3.当必须等待的时候，我总是心急如焚。	1	2	3
4. I never feel I have enough time.<br>4.我从未感到自己的时间宽裕。	1	2	3
5. I try to undertake multiple tasks at the same time.<br>5.我试图在同一时间内完成多件任务。	1	2	3
6. I always walk fast, even though there is nothing urgent to deal with.<br>6.即使没有什么要紧事，我走路也很快。	1	2	3
7. I am always eager to express my own opinion and even interrupt others when they are talking.<br>7.聊天时我总是急于说出自己的想法，甚至打断别人的话。	1	2	3
8. I always can't stand the shortcomings of others.<br>8.对别人的缺点和毛病，我常常不能容忍。	1	2	3
9. I always correct others' wrong opinions immediately.<br>9.听到别人发表不正确的见解，我总想立即就去纠正他。	1	2	3
10. When people are rude to me, I will retaliate immediately.<br>10.当别人对我无礼时，我会立即以牙还牙。	1	2	3
11. I am irritated easily.<br>11.我很容易发怒。	1	2	3
12. When I am busy doing something, I will be very annoyed if anyone disturbs me, whether he is intentional or not.<br>12.当我正在做事，谁要是打扰了我，不管有意无意，我都非常恼火。	1	2	3
13. When I was in line to buy something, if someone cut the line, I couldn't help blaming him or interfering.	1	2	3

13.排队买东西，要是有人加塞，我就忍不住指责他或出来干涉。			
14. I always spend more time on my work than necessary.<br>14.即使我的工作不需要花很多时间，我也总是花很长时间在工作上。	1	2	3
15. I feel that things I worry about are far more than I should.<br>15.有时连我自己都觉得，我所操心的事远远超过我应该操心的范围。	1	2	3
16. I often try to convince others to agree with me.<br>16.我常常力图说服别人同意我的观点。	1	2	3
17. I often feel anxious when a day has gone by without finishing my work.<br>17.我常常为工作没有做完，一天又过去了而感到忧虑。	1	2	3

Note: The “time urgency and impatience” dimension includes items 1-6, the “hostility and anger” dimension includes items 7-13, the “job engagement” dimension includes items 14 and 15, and the “competitiveness” dimension includes items 16 and 17.
